# Atypical Forms of Disease as Influenced by Environments

**Published:** 1893-08

**Authors:** L. M. Stroud

**Affiliations:** Terrell


For Texas Medical Journal.

### ATYPICAL FORMS OF DISEASE AS INFLUENCED BY ENVIRONMENTS.

BY L. M. STROUD, M. D., TERRELL.

[Read before the Terrell Medical Society and voted to Texas Medical Journal.]

THE study of types in nature is no less beautiful than instructive. The versatility of natural laws in modifying types in the animal, vegetable or bacteriological kingdom from environments has challenged the best thought of the scientific world during the last quarter century. Environments have subdivided species into multiform varieties in the animal, vegetable and bacteriological kingdom till the brief span of a life is insufficient to master all that is positively known in zoology, botany and bacteriology. Even the study of modifications of types of the same species as influenced by environments, furnishes a broad field to the scientific mind. To exemplify, the house cat is a species of the tiger changed by environments from its ferocious prototype of Bengal whose eyes glisten with thirst for blood to the innocent playmate and friend of the babe in the cradle.

The chin-oak, growing as a forest in miniature on the western border of Texas, and furnishing abundant mast for swine, is but the giant of the forest changed by environments.

The bacillus-malariæ of intermittent fever of our section is the same germ of pernicious intermittent which is the synonym of death in sub-tropical swamps, its natural home. Here the germ is so attenuated as to be amenable to a dose of quinine.

The bacillus-typhosis or so-called slow fever of our section is the same germ of malignant typhoid fever modified by environments as is attested in autopsies by the ulcerated Peyer's glands, as well as frequent deaths from hemorrhage of the bowels, and as is verified by the microscope. The disease being atypical in form, has given rise to the confusion of its nomenclature and consequent errors of diagnosis and treatment.

The sporadic form of diphtheria, originating in our section every year, is malignant epidemic diphtheria, modified by environments, the germ being so attenuated as to render the disease atypical in form. Its environments being unfavorable to the active proliferation of the specific bacilli, and consequently it is feebly infectious and incapable of originating an epidemic.

During a clinical observation of fourteen years in this section I have come in contact nearly every spring with a disease variously diagnosed, by local physicians, scarlet fever, rotheln, erythema, etc., and have witnessed two deaths among children in isolated situations in the country, where there had been no exposure to any known source of infection. By careful analysis of all the concomitant symptoms I am confirmed in the belief that it is simply an atypical form of scarlet fever.

In conclusion, I wish to refer to the influence of environments upon the tubercular bacilli. Every well informed physician is aware of the marvelous recovery of patients having incipient phthisis, going from parts of the country, the topography of which exposes the patient to a damp, cold atmosphere, to the high, dry plains of southwestern Texas and Mexico. The physical laws of nature insure in that section a dry, pure atmosphere and an abundance of solar temperature winter and summer. The transition from summer to winter being hardly appreciable, favoring an outdoor life of the patient in the sun and fresh air during the whole year. The environments of the climate being unfavorable to the life and propagation of the tubercular bacilli, enabling us, with a rational expectation of benefit to send our patients there in the hope they will outlive the disease.

Until we adopt the theory of modified germ life from environments as influencing disease, we will have no rational diagnosis or treatment in the atypical forms of typhoid fever, diphtheria, scarlet fever, besides many other clinical conditions we could mention, being unable not only to formulate a rational treatment in the management of these cases, but will be without the benefit of prophylactic measures in limiting them by suitable sanitary precaution.

